# Epigenetic regulation of human papillomavirus transcription in the productive virus life cycle

**DOI:** 10.1007/s00281-019-00773-0

**Published:** 2020-01-09

**Authors:** Megan Burley, Sally Roberts, Joanna L. Parish

**Affiliations:** College of Medical and Dental Sciences, Institute of Cancer and Genomic Sciences, University of Birmingham, B152TT, Birmingham, UK

**Keywords:** Papillomavirus, Epigenetics, Life cycle, Chromatin structure, Transcription

## Abstract

Human papillomaviruses (HPV) are a large family of viruses which contain a circular, double-stranded DNA genome of approximately 8000 base pairs. The viral DNA is chromatinized by the recruitment of cellular histones which are subject to host cell–mediated post-translational epigenetic modification recognized as an important mechanism of virus transcription regulation. The HPV life cycle is dependent on the terminal differentiation of the target cell within epithelia—the keratinocyte. The virus life cycle begins in the undifferentiated basal compartment of epithelia where the viral chromatin is maintained in an epigenetically repressed state, stabilized by distal chromatin interactions between the viral enhancer and early gene region. Migration of the infected keratinocyte towards the surface of the epithelium induces cellular differentiation which disrupts chromatin looping and stimulates epigenetic remodelling of the viral chromatin. These epigenetic changes result in enhanced virus transcription and activation of the virus late promoter facilitating transcription of the viral capsid proteins. In this review article, we discuss the complexity of virus- and host-cell-mediated epigenetic regulation of virus transcription with a specific focus on differentiation-dependent remodelling of viral chromatin during the HPV life cycle.

## Introduction

At the time of writing, the *Papillomaviridae* (PV) family of viruses is composed of over 450 distinct types of human papillomavirus (HPV) and over 200 animal papillomaviruses (http://pave.niaid.nih.gov). Each PV type is defined by a > 2% difference in sequence from any other know type. PV types are arranged into distinct genera that share > 60% identity in the L1 open reading frame (ORF). HPV types are phylogenetically arranged in five genera; alpha, beta, gamma, mu and nu [1]. All known HPV types occupy a tightly defined niche; they exclusively replicate in keratinocytes within squamous epithelia of either the cutaneous or mucosal surfaces of the human body. Infection with the vast majority of HPV types results in benign disease that is often sub-clinical, but can develop into the growth of papillomas or warts at the epithelial surface. A subset of HPV types (HPV16, 18, 31, 33, 35, 39, 45, 51, 52, 56, 58, 59 and 66) are the causative agent of cancers of the anogenital and oropharyngeal tracts and defined as group I carcinogens by the World Health Organization [2]. Due to their association with cancer development, these so called high-risk HPV types have been most widely studied and will therefore be the focus of this review.

### HPV genome structure

**Fig. 1:**
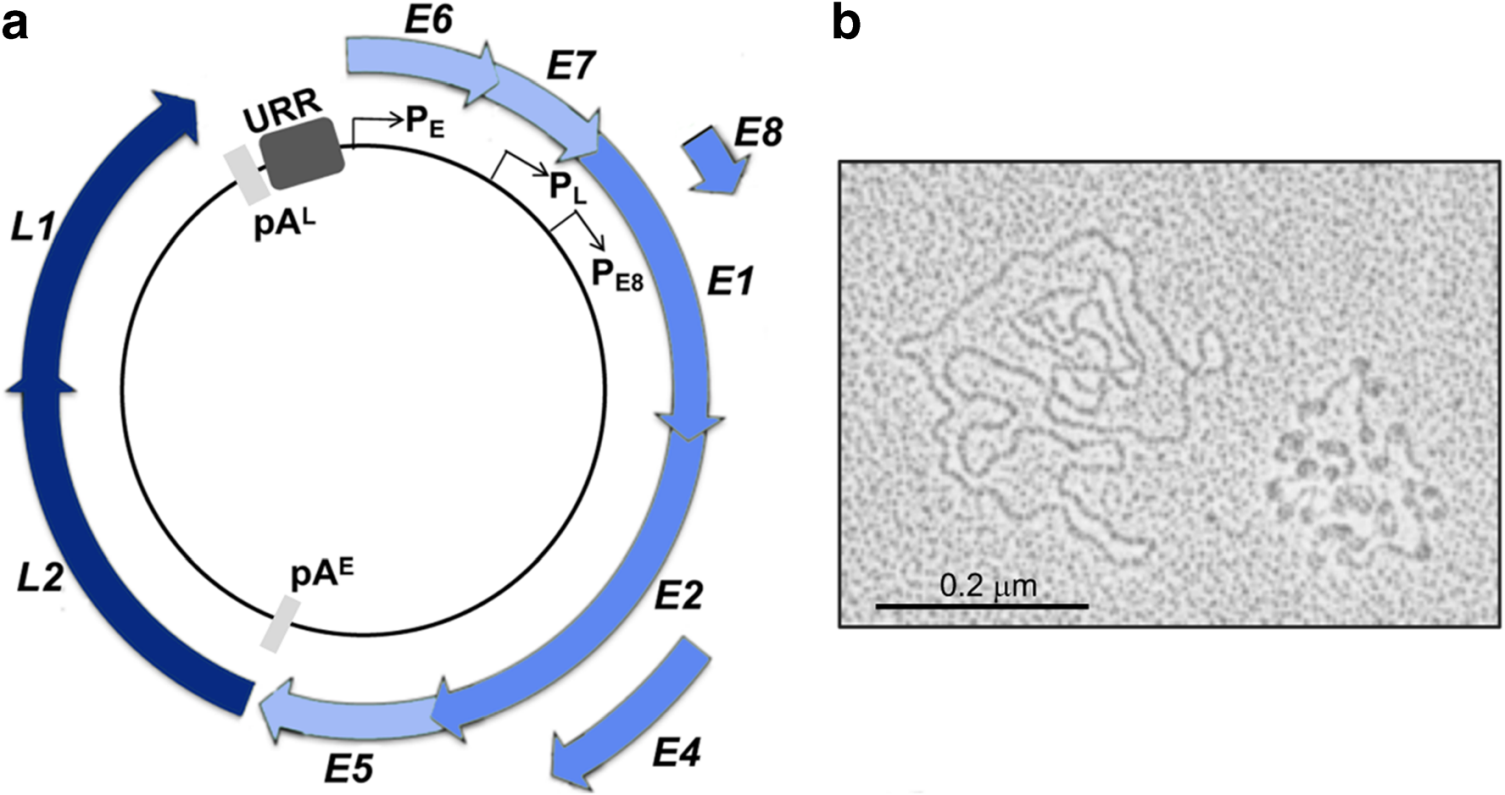
HPV genome organization. **a** The circular, double-stranded HPV genome is about 8000 base pairs and divided into three regions: the early, late and non-coding upstream regulatory region (URR). The early region contains open-reading frames (ORF), some overlapping of E1, E2, E4, E5, E6, E7 and E8. The late region contains L1 and L2 ORF—the capsid proteins. Viral transcription is controlled by the HPV E2 protein and host factors binding sequences within the URR. The main promoters are the early P_E_, the late P_L_ and the E8 promoter P_E8_, and viral transcripts terminate at the early pA^E^ or late pA^L^ poly-adenylation sites. The URR also contains the origin of replication. **b** In a study in the late 1970s [3], electron microscopy of metal-shadowed HPV genomes isolated from plantar warts showed naked HPV DNA molecules (left) and nucleoprotein-DNA complexes (right) revealing an intricate ‘beads on a string’ conformation of nucleosomes. Reproduced with permission from the American Society for Microbiology

The genome of all HPV types has a similar arrangement characterized by an approximately 8000 base pair circular doubled-stranded DNA genome encased in a non-enveloped icosahedral capsid of about 55 nm in diameter [1]. The viral genome contains 7–9 open reading frames (ORF) divided into early (E1, E2, E4, E5, E6, E7 and E8, although E5 and E8 ORF are not present in the genomes of all HPV types) and late (L1 and L2) genes (Fig. 1a). The core proteins, E1 and E2, have key roles in viral DNA replication and amplification, and regulating viral transcription, and the L1 and L2 proteins form the capsid, as well as L2 having a role in delivery of the viral genome to the nucleus upon infection and viral genome encapsidation during capsid assembly. Accessory proteins include E4, E5, E6 and E7 and these proteins facilitate the different stages of the vegetative virus life cycle primarily by forming virus-host interactions to alter the environment of the keratinocyte to support viral replication and enable evasion of host anti-viral defences. For the high-risk HPV types, the key players in oncogenesis are the oncoproteins E5, E6 and E7. A non-coding region referred to as the upstream regulatory region (URR; also known as the long control region (LCR)) is situated upstream of the early region (Fig. 1a). This region contains binding sites for a plethora of transcription and regulatory factors that either activate or repress the early (E) and late (L) promoters (P_E_: P97 - HPV16, P105 - HPV18; P_L_: P670 - HPV16; P811 - HPV18), the origin of replication to which the E1 protein binds, as well as multiple binding sites for the viral E2 protein. Relevant to this review was a study in the 1970s that showed that the HPV genome does not exist in a naked state in an productive lesion but as a nucleoprotein complex containing cellular histones [3](Fig. 1b).

### HPV transcription

Several promoters within the HPV genome have been identified, but for the purposes of this review, we will mention here only the early promoter (P_E_) that is active in undifferentiated basal keratinocytes, the late promoter (P_L_) that lies within the E7 ORF and becomes active in differentiated keratinocytes, and the P_E8_ promoter present within the E1 ORF which controls the expression of an E8^E2 protein that regulates viral transcription and viral DNA replication. Alternative RNA splicing leads to the production of multicistronic viral messenger RNAs transcribed from one strand of the genome. Early transcripts initiate from P_E_ and terminate at the early polyadenylation (PolyA) site (PA^E^) situated at the end of the E5 ORF (Fig. 1a). Transcripts from the differentiation-regulated P_L_ also utilize the early PolyA site but those that encode the structural proteins terminate at the late PolyA motif (PA_L_) present in the URR. The P_E8_ promoter is active throughout the infectious cycle and unlike P_E_ and P_L_, constitutive activation of P_E8_ is not controlled by the viral enhancer elements situated within the URR [4].

The programme of HPV transcription is intimately linked to the physiology of the keratinocyte and all stages of RNA metabolism are regulated during the virus life cycle, including promoter usage, polyadenylation, splice site usage, mRNA stability and translation (reviewed in [5]). The overall effect of this complex, differentiation-specific programme ensures that low levels of those early proteins necessary for initial amplification and establishment of the viral genome are expressed in basal cells. As differentiation occurs and the life cycle switches to the vegetative cycle, the expression levels of these proteins rise along with E4, E5, E6 and E7 to alter the keratinocyte milieu to enable viral DNA amplification and restrict expression of the structural proteins necessary for virion assembly in the upper most differentiated cells. One key aspect of this programme is that it avoids high expression levels of the viral proteins in basal keratinocytes and thereby avoids activation of host immune pathways. Integral to this control process of HPV transcription is epigenetic modification of the viral chromatin.

### HPV life cycle

**Fig. 2:**
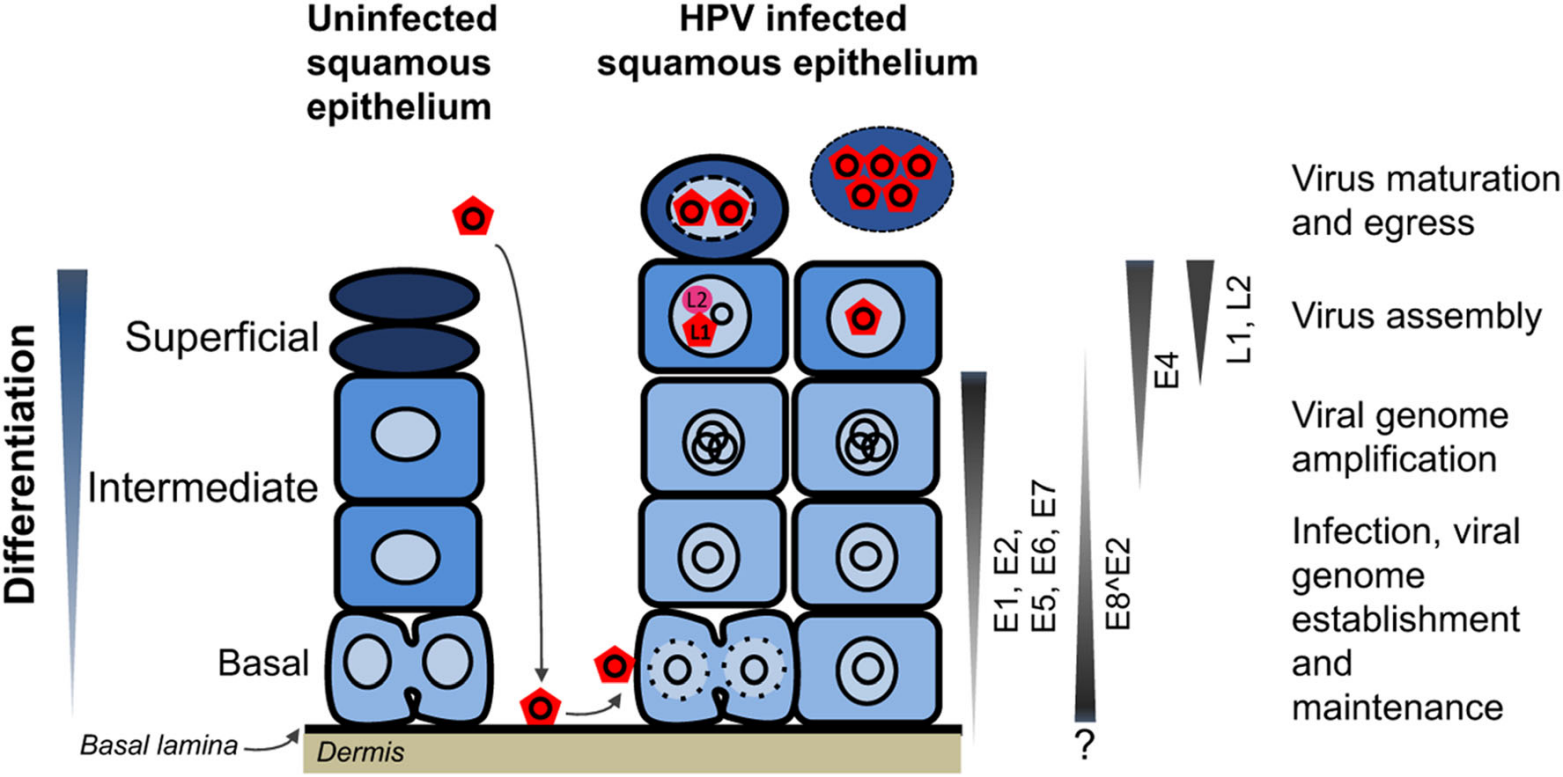
HPV life cycle organization. The HPV life cycle is dependent on the terminal differentiation of the keratinocyte. The virus life cycle begins by the viral particle (red pentagon) gaining access to the basal lamina and then infecting the mitotically active basal keratinocyte. The viral genome (black circle) is established as an extra-chromosomal replicon and maintained in basal cells until the cell differentiates and HPV early protein expression increases. Differentiating cells are pushed back into cell cycle and the viral genome amplifies to high copy number. Finally, the cell completes differentiation, expresses the viral late structural proteins L1 and L2 enabling virion assembly and egress. A viral regulator E8^E2 regulates viral transcription and replication and can also inhibit its own promoter P_E8_ suggesting that levels of the regulator may be finely tuned during the life cycle

**Fig. 3:**
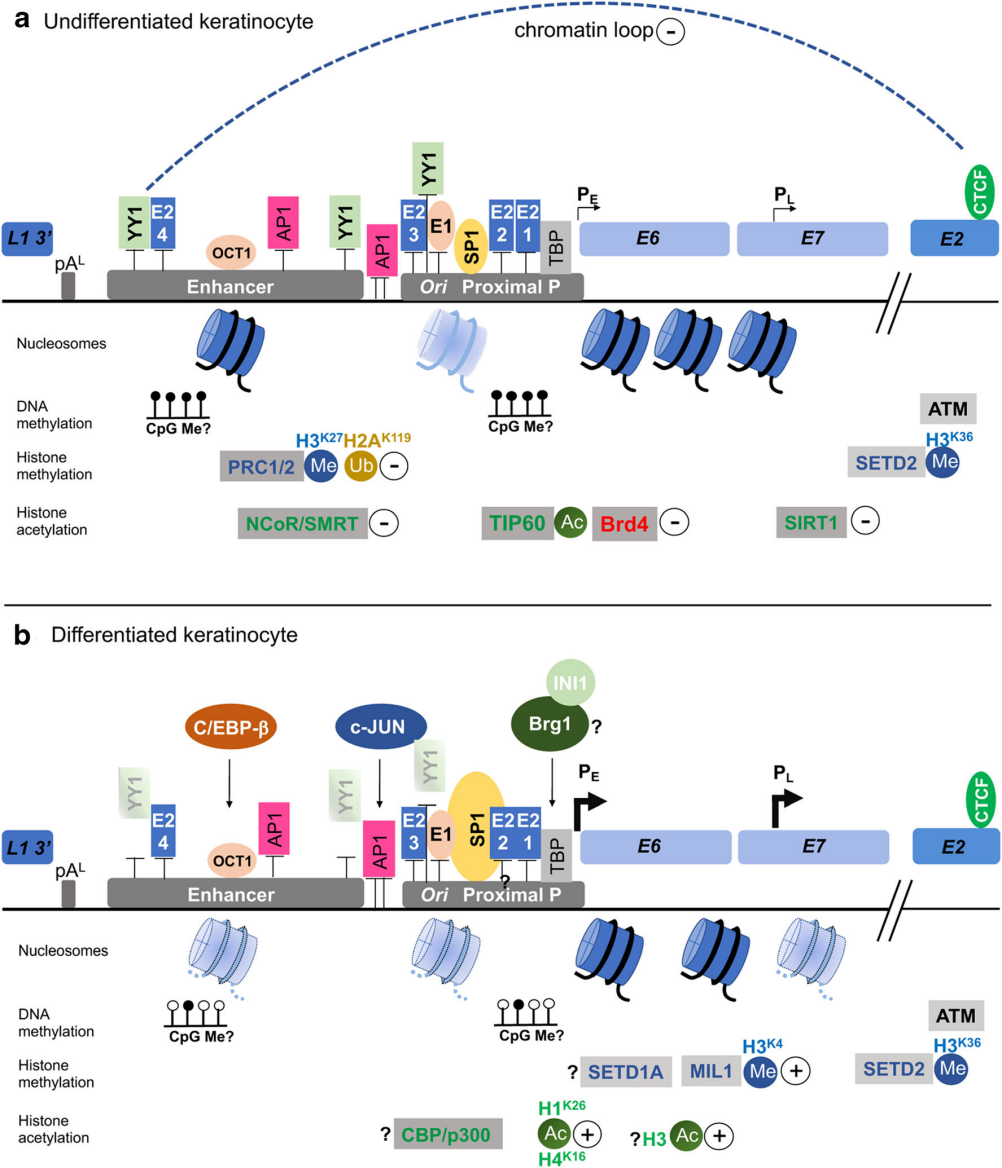
Epigenetic regulation of the HPV transcription during the virus life cycle. Binding of nucleosomes to the HPV URR (contains the enhancer, origin of replication (*Ori*) and proximal promoter P_E_), epigenetic modifications (DNA methylation (CpG Me), histone methylation (Me) and histone acetylation (Ac)) and some of the major host epigenetic modifiers involved are shown in undifferentiated (**a**) and differentiated keratinocytes (**b**). Details are given in the text. Strongly bound nucleosomes are shown in dark blue, weakly bound in faint blue, and nucleosomes that are lost or repositioned upon differentiation are shown in faint blue and with dotted lines. Question marks (?) indicate uncertainty of the epigenetic modification occurring in differentiated cells. Plus and minus signs indicate stimulation or repression of viral transcription. Host factors known to bind the URR that are relevant to this review are shown only, alongside E1 and E2 binding

HPVs infect basal keratinocytes, the proliferative compartment of squamous epithelia, through wounds and micro-abrasions in the epithelium (Fig. 2). Keratinocyte infection is a lengthy multi-step cascade of host factor binding and protease-induced capsid conformational changes initiated following virion binding to heparin sulphate proteoglycans on the basal lamina. Upon mitosis of the infected keratinocyte, the incoming viral genome in complex with the minor capsid protein L2 is bound to the condensed chromatin. Following an initial phase of viral DNA amplification, the episomal genome is established at a copy number of approximately 50 to 100 copies per cell. The early proteins E1 and E2 along with host replication factors including DNA polymerase α/primase, replication protein A and topoisomerase I facilitate viral DNA replication; E1 functioning as an ATP-dependent DNA helicase to unwind the double-stranded DNA and E2 acting as a sequence-specific DNA binding protein to load E1 helicase onto the viral origin of replication in the URR (Fig. 3a).

The maintenance phase of HPV DNA replication occurs in synchrony with the host DNA which ensures equal partitioning of the viral episomes to daughter cells. E2 plays a central role in this process by tethering the viral genomes to host chromatin to ensure efficient inheritance of the viral DNA. The viral genomes are tethered to different regions of mitotic chromosomes and this involves interaction of E2 with different host factors, including the epigenetic reader bromodomain protein Brd4, the DNA helicase chromosome loss-related 1 (ChlR1) and topoisomerase binding protein 1 (TopBP1) (reviewed in [6]). E2-mediated tethering via Brd4 association also appears to be important for positioning viral genomes to host genomic fragile sites that contain large and actively transcribed genes in epigenetically active chromatin [7].

E2 protein function is also central to virus transcription control. There are four conserved palindromic E2 binding sites within the URR of high-risk alpha-HPVs with the consensus sequence ACCG(N)_4_CGGT that each binds an E2 dimer [8] (Fig. 3a). Binding of E2 to these sites can either activate or, more commonly, repress transcription depending on the sequence context of the occupied E2 sites within the URR. The mechanism of E2-dependent transcriptional repression is both through steric hindrance of transcriptional activators such as TATA binding protein (TBP) and specificity protein 1 (SP1) to proximal promoter elements [9–13] or by facilitating recruitment of cellular factors that mediate epigenetic regulation of the viral chromatin [14–16].

The E8^E2 protein is encoded by a transcript that originates from the P_E8_ promoter situated within the E1 ORF of many but not all animal and human papillomavirus types. The E8^E2 product is formed from an alternative exon within the E1 ORF (E8) spliced into the 3′ exon of the E2 gene such that E8^E2 protein contains a novel N-terminal domain fused to the DNA-binding domain of E2 [17]. As such, E8^E2 is unable to bind to the E1 helicase and many of the cellular transcriptional regulators bound by the N-terminal domain of full length E2 but retain the ability to homo- and hetero-dimerize with full length E2 and bind to E2 DNA binding sites in the viral URR [18]. E8^E2 is therefore a strong repressor of HPV replication by excluding E1 from the origin of replication, and of E2-dependent transcription activation by preventing the recruitment of E2-associated transcriptional activators to the URR. Although P_E8_ is constitutively active and independent of enhancer elements with the HPV URR, E8^E2 is able to strongly inhibit its own promoter but E2 weakly activates it. Such fine tuning of E8^E2 expression requires E2/E8^E2 binding within the URR and is thought to represent a mechanism whereby HPV controls viral copy number [4].

Once the infected cell moves from the basal lamina, the normal entry into differentiation is delayed and early gene expression increases with stimulation of P_E_ and P_L_ activities. E5, E6 and E7 protein functions synergize to promote cell cycle re-entry and proliferation, and survival of post-mitotic keratinocytes to orchestrate viral DNA replication competent cells. They do so by targeting the components of key growth control pathways including p53 and retinoblastoma, as well as pathways that enable the virally infected cells to avoid immune detection [19]. The virus also deregulates epigenetic control of host gene expression contributing to the proliferative state and survival of the infected cell [20]. To avoid competing with host DNA replication during S phase, viral DNA amplification occurs in the G2 phase of the cell cycle, and by activating the ATM DNA damage repair pathways, HPV utilizes the repair factories that form to replicate its genome during this phase [21]. Once viral DNA amplification is completed, E2 binds to sites within the URR to repress the expression of early proteins and cell cycle activity ceases, enabling keratinocyte differentiation and the expression of the late structural proteins L1 and L2. This late productive stage encompasses viral genome encapsidation, maturation of progeny virus and the sloughing off of superficial cells packed with infectious new progeny from the epithelial surface. The vegetative phase is accompanied by expression of large quantities of E4, a viral protein of ill-defined function but one that contributes to the efficiency of this phase of the virus life cycle [22].

## Epigenetic chromatin organization

### Histone modification

DNA in the nucleus of eukaryotic cells is wrapped around octameric complexes of proteins called histones, composed of dimers of H2A, H2B, H3 and H4. Each histone core is wrapped by ~ 147 base pairs of DNA [23] to form nucleosomes that create a classical ‘beads-on-a-string’ conformation known as chromatin. Chromatin structure is central to the control of gene transcription as the arrangement of nucleosomes on regulatory units controls accessibility to transcription factors. Histones can be covalently modified on the N-terminal tails that protrude from the core histone complexes by a series of enzymes known as writers including DNA methyltransferases (DNMTs), histone lysine methyltransferases, protein arginine methyltransferases and histone acetyltransferases (HATs). Such modifications include mono- (Me1), di- (Me2) and tri- (Me3) methylation, hydroxy-methylation and acetylation (Ac) which are added to a plethora of arginine (R) and lysine (K) residues within the protruding N-terminal tails of histones. In altering the affinity of histones for DNA, specific covalent modification of histones can differentially recruit or disrupt the binding of factors termed readers that further regulate chromatin structure and function, such as remodelling enzymes that drive repositioning of histones (for review see [24]).

Through epigenetic modification, chromatin exists in different physical states to regulate transcription. Tightly packed, closed chromatin known as heterochromatin is transcriptionally repressed, whereas open chromatin known as euchromatin is permissive for gene transcription as it contains much less densely packed and dynamically associated histones allowing transcription factors to access regulatory elements and drive transcription. These general forms of chromatin are dictated by post-translational modification (PTM) of histones or by direct, covalent methylation of DNA. Heterochromatin is generally characterized by enrichment of repressive epigenetic marks such as H2K4Me2/3, H3K9Me1, H3K27Me2/3 and H4K20Me3. Acetylation of histones decreases their affinity to DNA and as such open euchromatin is enriched in epigenetic marks including H3K4Ac and H3K27Ac [25]. The diversity of histone PTMs that regulate the epigenome creates a gradient of chromatin structure ranging from heterochromatin to repressed but permissive chromatin that can be rapidly activated to constitutively active chromatin. Reversible changes to chromatin that influence gene expression were first hypothesised in 1942 [26]. Evolution of this initial hypothesis over many decades of research has now defined epigenetics as heritable changes occurring in the genome that regulate gene expression patterns without affecting the underlying DNA sequence. Epigenetic regulation of gene expression is crucial in cellular programming during development and in the regulation of cellular processes and response to environmental stimuli without altering the underlying genetic code.

### DNA methylation

DNA can be directly methylated on the 5′ position of the cytosine pyrimidine ring creating 5-methylcytosine (5mC). This covalent modification most often occurs on cytosines preceding a guanine (CpG), and is catalysed by DNA methyltransferases (DNMTs) which catalyse either maintenance or de novo DNA methylation (reviewed by [27]). While CpG methylation occurs globally across the genome, there are large clusters of these sites, known as CpG islands [28]. CpG islands are important in regulating chromatin structure and gene expression control. Up to 60% of gene promoters contain CpG islands in which methylation blocks transcription initiation. However, methylation within gene bodies can also enhance transcription and alter gene splicing [29]. DNA methylation regulates gene silencing via a number of mechanisms. It can mediate the direct inhibition of essential protein-DNA interactions and reduce chromatin accessibility [30]. CpG methylation is also known to recruit methyl-CpG binding proteins (MeCPs), resulting in further alteration of chromatin structure [31]. Cytosine methylation is mediated by three key members of the DNMT family which possess methyltransferase activity. The activity of DNMT1 is preferential for hemi-methylated DNA and is often referred to as a maintenance methyltransferase while the DNMT3 family (DNMT3A, DNMT3B) can also catalyse de novo DNA methylation.

## Epigenetic regulation of HPV transcription

### HPV chromatin structure

The association of histone complexes with encapsidated HPV DNA was first described by Favre and colleagues in 1977 [3]. Electrophoresis of highly purified HPV virions revealed association with proteins of similar molecular mass to the canonical histone complex, H2A, H2B, H3 and H4, and it was estimated that these histone-like proteins constituted 87% of the total DNA-associated protein. Nucleated HPV DNA was analysed by electron microscopy which revealed an intricate ‘beads on a string’ conformation with each nucleosome measuring 12 nm in diameter corresponding to canonical nucleosomal formation. Up to 32 nucleosome complexes were observed on the complete HPV genome and interestingly, the interconnecting DNA was of variable length indicating sequence- or regulatory element–dependent positioning of nucleosomes (Fig. 1b). The precise arrangement of nucleosomes on the viral enhancer and promoter elements is likely to be fundamental to virus transcription regulation. Nucleosome mapping demonstrates that at least two nucleosomes are located within the URR in HPV16 and 18, one overlapping with the viral enhancer and a second overlapping with the E1 binding site within the replication origin and SP1 binding site in the early promoter [32] (Fig. 3a). The nucleosome positioning at the early promoter functions to repress virus transcription by excluding SP1 recruitment [32]. However, the replication origin and early promoter have been shown to have weaker affinity for histones than other areas of the viral genome [33], suggesting that this nucleosome is easily displaced to activate transcription and/or replication. Increased SP1 concentration can displace this nucleosome in vitro [32]. The E1 and E2 proteins have also been shown to induce a change in nucleosomal positioning [33, 34] suggesting that nucleosome arrangement is dictated by DNA sequence as well as the binding of host and viral factors. Further three nucleosomes are positioned at the late promoter within the E6 ORF and 5′ end of the E7 ORF [32, 35] (Fig. 3a). Interestingly, significant remodelling of chromatin structure with the E7 ORF occurs upon keratinocyte differentiation to increase accessibility and activation of the late promoter [35] (Fig. 3b).

### Histone acetylation

HPV has been shown to interact with several HAT and HDAC family members to regulate viral transcription. CREB-binding protein (CBP) and its paralogue p300 are transcriptional coactivators that bind DNA-bound transcriptional regulators and acetylated histones. Once bound to a promoter, CBP/p300 recruit the basal transcription machinery to activate transcription. CBP/p300 also have intrinsic HAT activity and can acetylate histones [36], thereby causing relaxation of DNA at transcriptional promoters, and basal transcription factors to further activate transcription [37]. Numerous studies have demonstrated a role for p300 in maintaining the high expression of E6/E7 in cervical cancer cells. The E2, E6 and E7 proteins from various HPV types have all been shown to bind to p300 [38–41]. HPV E2 and p300 cooperate to activate the HPV early promoter cloned into transcriptional reporter constructs [42] and the interaction between E7 and p300 may be an important feedback loop as E7 abrogates CBP/p300-mediated E2 transactivation [41]. CBP/p300 can also bind to the HPV18 URR in the absence of E2 as recruitment has been demonstrated in E2-negative cervical cancer cells [43]. CBP/p300-dependent E6/E7 transcription activation is associated with acetylation of H3 at the HPV URR providing evidence that CBP/p300 activates HPV transcription by altering the epigenetic status of the viral enhancer/promoter [44]. Increased histone acetylation by CBP/p300 results in enhanced recruitment of the SWI/SNF chromatin remodelling complex catalytic subunit, the Brahma-related gene-1 (Brg1), to the URR which is required for efficient RNA polymerase II recruitment [45]. Interestingly, chemical inhibition of p300 HAT activity inhibits E6/E7 mRNA expression and induces apoptosis cervical cancer cells [46], suggesting that CBP/p300 inhibition may be an effective anti-HPV strategy. While these studies demonstrate a role for CBP/p300 in the sustained E6/E7 expression in HPV-driven cancer, the function of HAT activity in the productive virus life cycle is not understood although increased histone acetylation has been detected at the URR and late promoter following host cell differentiation.

In the context of an HPV infection, E2 functions to repress E6/E7 transcript production. In an siRNA screen designed to identify cellular factors that contribute to E2-mediated repression of the HPV18 URR, EP400, a component of the NuA4/TIP60 histone acetylase complex was identified [15]. Acetylation of histones in the HPV URR by TIP60 results in the recruitment of bromodomain containing protein Brd4 [47]. Brd4 is a strong corepressor of E2-dependent HPV transcription [15, 16]. Therefore, rather than functioning as a coactivator of transcription as is the canonical function of TIP60, recruitment of TIP60 to the URR results in Brd4 recruitment and strong transcriptional repression.

Sirtuins (SIRT1-SIRT7) are a protein family of class III HDACs that function in DNA damage repair and apoptosis. The stable maintenance of HPV16 and HPV31 episomes within human foreskin keratinocytes results E6/E7-dependent elevation of SIRT1 expression. This increase is maintained within differentiated keratinocytes [48]. SIRT1 promotes HPV episome replication in undifferentiated keratinocytes and genome amplification upon differentiation and is important for late transcription production in differentiated cultures [48, 49]. In undifferentiated cells, SIRT1 binds to the HPV31 URR and deacetylates histone 1 at Lys26 (H1K26Ac) and histone 4 at Lys16 (H4K16Ac), enabling repression of late gene transcription. SIRT1 also stimulates the recruitment of Werner helicase (WRN) to enhance E1-E2-dependent replication fidelity [50]. Following differentiation, SIRT1 binding to HPV episomes is significantly reduced resulting in the hyperacetylation of histone-1 (Lys26) and enhanced late gene expression [48]. Interestingly, SIRT1 knockout results in reduced E2 protein acetylation suggesting that E2 is a direct target for SIRT1 [49]. Further epigenetic repression of the viral URR is mediated by E8^E2-mediated recruitment of the HDAC3-containing NCoR/SMRT transcriptional repressor complex [51].

### Histone methylation

The viral episome in undifferentiated keratinocytes exists in a repressed chromatin state in part by the recruitment of polycomb repressor complexes 1 and 2 (PRC1/2) which catalyse deposition of repressive H3K27Me3 and H3K119Ub [52]. While this is likely to be important for the productive virus life cycle, integration of viral DNA and upregulation of viral oncogene E6/E7 expression have been shown to correlate with enrichment of open chromatin at the HPV16 LCR and early promoter, mediated by chromatin remodelling enzymes Brg1 and INI1 (hSNF5/SmarcB1) [53]. This disease-associated alteration of the epigenetic status of HPV chromatin increases the accessibility of positive transcriptional regulators including c-Jun and histone lysine methyltransferases, including SETD1A and MIL1, which catalyse deposition of transcriptionally active histone marks, including H3K4me3 creating a favourable landscape for RNA polymerase II recruitment which drives HPV16 oncogene transcription from the early promoter [53]. Whether this is important in the productive virus life cycle has yet to be determined.

The histone methyltransferase SETD2 is a writer of trimethylation of histone 3 lysine 36 (H3K36me3), a mark of active transcription. High-risk E7 mediates the post-transcriptional stabilization of SETD2 resulting in increased levels in HPV31 and HPV16 containing human foreskin keratinocytes. SETD2-dependent H3K36me3 deposition is apparent throughout the viral genome and enriched at the 3′ end of the early gene region in both undifferentiated and differentiated keratinocytes and is essential for both maintenance and productive viral replication [54]. Interestingly, the DNA damage kinase enzyme ataxia-telangiectasia mutated (ATM) is required for maintenance of H3K36Me3 on viral chromatin presumably through inhibition of the demethylases KDM2A and/or KDM4A suggesting that ATM not only facilitates recruitment of DNA damage repair factors to the viral genome but also influences epigenetic status [54]. Conversely, HPV E7 has been shown to enhance cellular expression of the H3K27Me3 demethylase KDM6A, resulting in de-repression of host genes [55] but the consequences of KDM6A upregulation on the epigenetic status of the viral genome have not been studied.

### CpG DNA methylation

The first evidence of epigenetic modification of HPV DNA was in the form of covalent methylation of CpG dinucleotides on HPV1 DNA [56, 57]. It was initially demonstrated that CpG methylation of integrated HPV18 DNA in tumourigenic and non-tumourigenic cell lines has an inverse correlation with virus transcript levels. In addition, treatment of HeLa cells with the DNA methylation inhibitor 5-azacytidine resulted in reduced HPV mRNA expression [58]. Purified HPV18 DNA can be CpG methylated in vitro resulting in attenuation of activity of transfected HPV transcription reporters [59]. Differentially methylated CpG dinucleotides are present within consensus E2 binding sites in the URR and CpG methylation at these sites inhibits E2 binding, alleviating E2-mediated repression of E6/E7 oncogenes [60]. CpG methylation changes that are initiated by cellular differentiation may influence E2-dependent virus transcription during the HPV life cycle although this is not understood. Studies in HPV16-episome containing W12 cells derived from a naturally occurring low-grade cervical lesion [61] demonstrated that the viral LCR is enriched in methylated CpG dinucleotides in poorly differentiated cells and become hypomethylated upon cellular differentiation [62]. Although it has also been noted that episomal HPV DNA in premalignant biopsy material is unmethylated suggesting that de novo methylation may occur after integration of HPV DNA into the host to attenuate production of viral transcripts, which could result in viral latency [59]. Transcriptionally silent HPV integrants can be found in the healthy cervices of older women suggesting that such a mechanism of HPV latency may be at play [63].

Modulation of CpG methylation of HPV DNA is important during carcinogenesis. Several studies have demonstrated a correlation between increased CpG methylation within the late gene region of integrated viral sequences and disease progression [64–67]. High-grade cervical intraepithelial neoplasia (CIN2+) cases show significantly higher methylation compared with HPV DNA clearance controls and this was found to be largely associated with the L1 and L2 ORFs [65]. Interestingly, a correlation between increased methylation status of E2 binding sites in the URR in the presence of an intact E2 ORF and disease severity has been reported in oropharyngeal squamous cell carcinomas (OPSCC) [68]. Since the DNA binding affinity of E2 is reduced by CpG methylation, this is likely to explain why CpG methylation of the HPV URR correlates with increased E6/E7 expression compared with tumours with undetectable methylation [60, 68], and that high-grade OPSCC frequently have high E2 expression combined with high E6/E7 expression [69].

## Differentiation-dependent regulation of the HPV epigenome

Stimulation of keratinocyte differentiation results in an increase in transcripts that originate from the early protomer and the appearance of transcripts that are initiated from within the E6/E7 ORFs around the late promoter [35, 70, 71]. Sequences within the URR and E6/E7 gene regions are required for late promoter activation [72] and it was proposed that differentiation-dependent regulation of HPV transcription was initiated by changes to chromatin structure in these regions enhancing accessibility to host transcriptional regulators. DNase I footprinting experiments identified a region within the E7 ORF that was depleted of histone proteins following differentiation of HPV31 episome harbouring keratinocytes [35]. Differentiation-induced enhancement of chromatin accessibility was shown to be due to alterations in epigenetic status of the viral chromatin including dramatic enhancement of H3 and H4 acetylation and H3K4Me2 at both the early and late promoters [73]. These changes in chromatin structure are co-incident with enhanced binding of cellular transcriptional activators such as C/EBP-β and c-Jun to the keratinocyte specific enhancer within the URR [73].

YY1 is a strong repressor of the HPV keratinocyte specific enhancer [74, 75] and functions as a transcriptional repressor by facilitating the recruitment of the Polycomb group (PcG) of proteins to chromatin [76–78]. PcG proteins are epigenetic writers that assemble into multimeric complexes including the Polycomb repressive complexes 1 and 2 (PRC1, PRC2). PRC1 functions as a ubiquitin ligase which specifically ubiquitinylates H2A lysine 119 (H2AK119Ub) [79]. PRC2 contains the methyltransferase Enhancer of Zeste homologue 2 (Ezh2) which catalyses transcriptionally repressive H3K27Me3 deposition [80]. PRC1 and PRC2 are enriched on HPV18 URR in undifferentiated keratinocytes coincident with the enrichment of H3K27Me3 and H2K199Ub, and repression of virus transcription [52]. Studies have shown that expression of E6 and E7 from HPV types 16, 18 and 38 induces increased Ezh2 protein levels, which was shown to be required for the proliferation of HPV-positive tumour cells although a concomitant increase in H3K27Me3 levels was not observed [81–83]. This apparent disconnect is thought to be due to enhanced phosphorylation of Ezh2 at serine 21 induced by E6/E7 expression [82], which inhibits the enzymatic activity of Ezh2 [84]. It would be interesting to determine whether this represents a positive feedback mechanism of HPV transcription activation resulting from differentiation-induced enhancement of E6/E7 expression.

Chromatin binding architectural proteins such as the zinc-finger CCCTC-binding factor CTCF are fundamental in the three-dimensional organization of chromatin. CTCF is a ubiquitously expressed DNA-binding protein that binds tens of thousands of sites in the human genome [85] and functions as an epigenetic boundary insulator, transcriptional activator and repressor [86]. By facilitating the formation of chromatin loops at sites that are also enriched in cohesin [87], CTCF is important in the maintenance of long-range chromatin interactions [88]. Interestingly, the majority of genomic chromatin loops are stabilized by CTCF bound to inverted cognate sites [89] and inversion of specific CTCF sites has profound effects on chromatin loop formation [90]. The specificity of CTCF binding site orientation has been central to the hypothesis that chromatin loops are formed by extrusion of the DNA through cohesin rings that are blocked by CTCF ‘anchors’ at specific genomic loci. Whether there is a specific motor complex that drives chromatin loop extrusion is yet to be decided, but theoretical modelling suggests that loop extrusion may occur via diffusive motion within the nucleus rather than an ATP-dependent motor protein [91].

CTCF has been demonstrated to regulate transcription of several large DNA viruses including the γ-herpesviruses Kaposi sarcoma-associated herpesvirus (KSHV) and Epstein-Barr virus (EBV) [92–96]. Similarly, multiple CTCF binding sites have been identified within the genomes of several HPV types. These include a conserved cluster within the late gene region found in over 80% of 125 types screened (high- and low-risk) and also sites within the E2 ORF, present in less than 20% of HPV types analysed and which appears to be conserved in high-risk HPV types only [97, 98]. Using HPV31 episome-containing cells as a model system, the Laimins group showed that CTCF was predominantly recruited to the late gene region and that depletion of CTCF, or mutation of the L2 binding site cluster resulted in reduced episome copy number and failure of episomal establishment [97]. Somewhat in contrast to these findings, our laboratory has shown that HPV18 genomes have enriched CTCF binding at the high-risk HPV-specific E2 ORF with an absence of binding in the late gene region, suggesting that different high-risk HPV types have evolved different strategies of gene expression control [52, 98]. Mutation of the single E2-CTCF binding site in HPV18 had no effect on replication or maintenance of HPV18 episomes, but resulted in increased early transcript production and a concomitant increase in E6 and E7 protein expression and cellular hyperproliferation [98]. Importantly, we showed that CTCF-mediated repression of HPV early gene transcription via the stabilization of a chromatin loop formed between the E2 ORF and the URR. Rather than being formed between two convergent CTCF binding sites, as has been shown in the host genome, the CTCF-dependent chromatin loop in HPV18 episomes is formed between CTCF bound at the E2 ORF and a second transcription factor, Yin Yang 1 (YY1), bound at the viral URR [52] (Fig. 3a). CTCF and YY1 have been previously shown to directly interact and co-operate in the stabilization of chromatin loops between distant loci in the human genome, providing an alternative mechanism of chromatin organization [99, 100].

It is feasible that loop formation in the HPV genome occurs via loop extrusion between the CTCF and YY1 anchor points but whether this loop is stabilized by cohesin is currently unknown although phosphorylated SMC1 (pSMC1), a structural component of cohesin rings, binds to HPV31 episomes that appears to be important for viral genome amplification [97]. Whether SMC1 also plays a role in HPV transcription and/or whether cohesin is required for chromatin loop stabilization has yet to be resolved. While YY1 is abundantly expressed in undifferentiated keratinocytes and recruited to the HPV URR, stimulation of keratinocyte differentiation causes a dramatic reduction in YY1 protein expression and a loss of YY1 recruitment to the URR (Fig. 3b). This causes a loss of repressive chromatin loop formation in the viral genome, stimulating increased early gene transcription [52]. Whether the loss of chromatin loop formation in HPV18 genomes during keratinocyte differentiation is directly responsible for late promoter activation is presently unknown.

Interestingly, ChIP-Seq data available from the ENCODE project [101] for HeLa cells have been analysed to define histone and transcription factor occupancy on the integrated HPV18 locus. While the CTCF binding site within the E2 ORF is maintained in the integrated HPV18 DNA, CTCF protein was not detected at this site [102]. This may indicate that the repression of viral transcription via CTCF-YY1 chromatin organization is abrogated in HPV-driven cancer cells, a hypothesis we are currently testing. Since CTCF binding sites have been shown to be a major hotspot for mutation in a variety of cancers [103], the loss of CTCF binding to viral DNA in tumours may represent a similar driving event in cancer development.

## Conclusions

Epigenetic regulation of HPV transcription is necessary for episome establishment, genome maintenance and completion of the productive HPV life cycle. The complex interplay of positive and negative epigenetic regulation of HPV transcription is inextricably linked to the differentiation status of the infected cell; the viral genome exists in an epigenetically repressed state in the undifferentiated basal cells with low-level gene expression such that the episome can replicate but prevent immune activation. As cells enter a programme of differentiation, epigenetic repression of the viral genome is alleviated and the viral chromatin structure is maintained in an active state, resulting in increased expression of viral replication proteins and activation of the late promoter and capsid protein production. This complex transcriptional programme, requiring a plethora of host cell epigenetic regulators, appears to be disrupted in HPV-induced carcinogenesis providing the possibility of new therapeutic strategies against HPV-induced disease.
